# Driving-pressure-independent protective effects of open lung approach against experimental acute respiratory distress syndrome

**DOI:** 10.1186/s13054-018-2154-2

**Published:** 2018-09-23

**Authors:** Kentaro Tojo, Tasuku Yoshida, Takuya Yazawa, Takahisa Goto

**Affiliations:** 1 Department of Anesthesiology and Critical Care Medicine, Yokohama City University Graduate School of Medicine, Yokohama, Kanagawa Japan; 2 Department of Pathology, Dokkyo Medical University, Tochigi, Japan

**Keywords:** Acute respiratory distress syndrome, Atelectasis, Driving pressure, Mechanical ventilation, Open lung approach, Platelet-derived chemokine, Positive end-expiratory pressure, Recruitment maneuver

## Abstract

**Background:**

The open lung approach (OLA) reportedly has lung-protective effects against acute respiratory distress syndrome (ARDS). Recently, lowering of the driving pressure (ΔP), rather than improvement in lung aeration per se, has come to be considered as the primary lung-protective mechanism of OLA. However, the driving pressure-independent protective effects of OLA have never been evaluated in experimental studies. We here evaluated whether OLA shows protective effects against experimental ARDS even when the ΔP is not lowered.

**Methods:**

Lipopolysaccharide was intratracheally administered to rats to establish experimental ARDS. After 24 h, rats were mechanically ventilated and randomly allocated to the OLA or control group. In the OLA group, 5 cmH_2_O positive end-expiratory pressure (PEEP) and recruitment maneuver (RM) were applied. Neither PEEP nor RM was applied to the rats in the control group. Dynamic ΔP was kept at 15 cmH_2_O in both groups. After 6 h of mechanical ventilation, rats in both groups received RM to inflate reversible atelectasis of the lungs. Arterial blood gas analysis, lung computed tomography, histological evaluation, and comprehensive biochemical analysis were performed.

**Results:**

OLA significantly improved lung aeration, arterial oxygenation, and gas exchange. Even after RM in both groups, the differences in these parameters between the two groups persisted, indicating that the atelectasis-induced respiratory dysfunction observed in the control group is not an easily reversible functional problem. Lung histological damage was severe in the dorsal dependent area in both groups, but was attenuated by OLA. White blood cell counts, protein concentrations, and tissue injury markers in the broncho-alveolar lavage fluid (BALF) were higher in the control than in the OLA group. Furthermore, levels of CXCL-7, a platelet-derived chemokine, were higher in the BALF from the control group, indicating that OLA protects the lungs by suppressing platelet activation.

**Conclusions:**

OLA shows protective effects against experimental ARDS, even when the ΔP is not decreased. In addition to reducing ΔP, maintaining lung aeration seems to be important for lung protection in ARDS.

**Electronic supplementary material:**

The online version of this article (10.1186/s13054-018-2154-2) contains supplementary material, which is available to authorized users.

## Background

Acute respiratory distress syndrome (ARDS) is a form of severe respiratory failure [1] caused by marked lung inflammatory responses during critical illness, such as severe infection, trauma, and burn [2]. The pathological features of ARDS are alveolar barrier disruption and leakage of protein-rich fluid into the alveolar spaces, causing pulmonary edema [2] and atelectasis [3, 4]. Atelectasis in ARDS not only causes respiratory failure, but also enhances lung injury through several mechanisms. First, in the atelectatic lung region, alveolar hypoxia induces inflammation [5, 6], pulmonary hypertension, and right cardiac failure [7]. Second, atelectasis exposes peri-atelectatic alveoli to repetitive alveolar collapse and reopening [8], and exacerbates the peri-atelectatic mechanical ventilation-induced stress [9, 10]. Third, development of atelectasis during mechanical ventilation increases the relative tidal volume and the (driving pressure) [11, 12], which induces overdistension of aerated lung regions. Therefore, reducing atelectasis is an important aspect in the lung-protective management of ARDS.

To reduce atelectasis, the open lung approach (OLA), including application of positive end-expiratory pressure (PEEP) and the lung recruitment maneuver (RM), has been introduced and has shown some clinical benefits [13–16]. Although several mechanisms have been speculated to underlie atelectasis-induced exacerbation of lung injury as described above, the reduction in ΔP has recently come to be considered as the primary lung-protective mechanism of OLA [17]. For example, some studies have indicated that the protective effects of OLA are mediated almost solely through decreases in ΔP [18, 19], suggesting that the primary goal in mechanically ventilated patients is lowering the ΔP, rather than improving lung aeration. However, it is unclear whether OLA has protective effects independent of lowered ΔP, because, in the previous experimental studies demonstrating the lung protective effects of OLA [8, 20–26], the same tidal volume was maintained in OLA-managed subjects as in control subjects. Consequently, ΔP in the OLA group was lower than that in the controls in these studies, because the relative tidal volume to aerated lung volume was lowered by OLA. We hypothesized that lowering ΔP alone would not be sufficient for lung-protective management and that aeration improvement would have protective effects when the ΔP is not lowered. Elucidating whether OLA has effects independent of ΔP can clarify the importance of lung aeration per se in the management of ARDS.

The aim of the present study was thus to elucidate whether OLA has protective effects against experimental ARDS in a rat model, compared to management without OLA, even when a similar ΔP was applied in both groups.

## Methods

### Animal experiments

All animal experimental protocols were reviewed and approved by the Animal Research Committee of Yokohama City University. Male Sprague–Dawley rats, aged 8−9 weeks, were used for all the experiments. The animals were housed under a 12-h light/dark cycle with food and water available ad libitum.

Rats were anesthetized with intraperitoneal injection of ketamine (50 mg/kg/body weight) and xylazine (5 mg/kg/body weight). Under general anesthesia, the trachea was exposed through a small incision at the anterior neck and 300 μL of lipopolysaccharide (LPS, *Escherichia coli* O111:B4, Sigma–Aldrich. St Louis, MO, USA) solution in phosphate-buffered saline (PBS) (5 mg/mL) was intratracheally administered through a 30-gauge needle. Rats were held in a 45° head-up position for 1 min after this instillation; thereafter, oxygen was administered for 30 min at a flow rate of 0.5 L/min. After recovery from anesthesia, rats were returned to their cages.

At 24 h after LPS administration, rats were again anesthetized with intraperitoneal ketamine and xylazine injection. The left femoral vein was cannulated and a 1:1 mixture of 1% propofol and acetate Ringer solution was injected at a rate of 1 mL/h. The right carotid artery was cannulated, and arterial blood pressure was monitored. From the arterial line, heparinized normal saline was injected at 3 mL/h. Thereafter, the trachea was cannulated and connected to a mechanical ventilator (SN-480-7, Shinano Seisakusho, Tokyo, Japan), which is designed for small animals. Rats were ventilated using the following parameters: fraction of inspired oxygen (F_I_O_2_), 0.4; tidal volume, 10 mL/kg; frequency, 65/min; and PEEP, 5 cmH_2_O. After starting mechanical ventilation, 0.4 mg of pancuronium bromide, followed by another 0.2 mg every 30 min, was administered to stop spontaneous breathing. Thereafter, the recruitment maneuver (RM) was performed, with 30 cmH_2_O for 10 s, three times. The Y-piece of the breathing circuit was connected to the pressure transducer (Becton Dickinson, Franklin Lankes, NJ, USA) by an air-filled pressure-resistant tube, and the airway pressure was recorded using a medical bedside monitor (BSM-8500, Nihon Kohden, Tokyo, Japan).

**Fig. 1:**
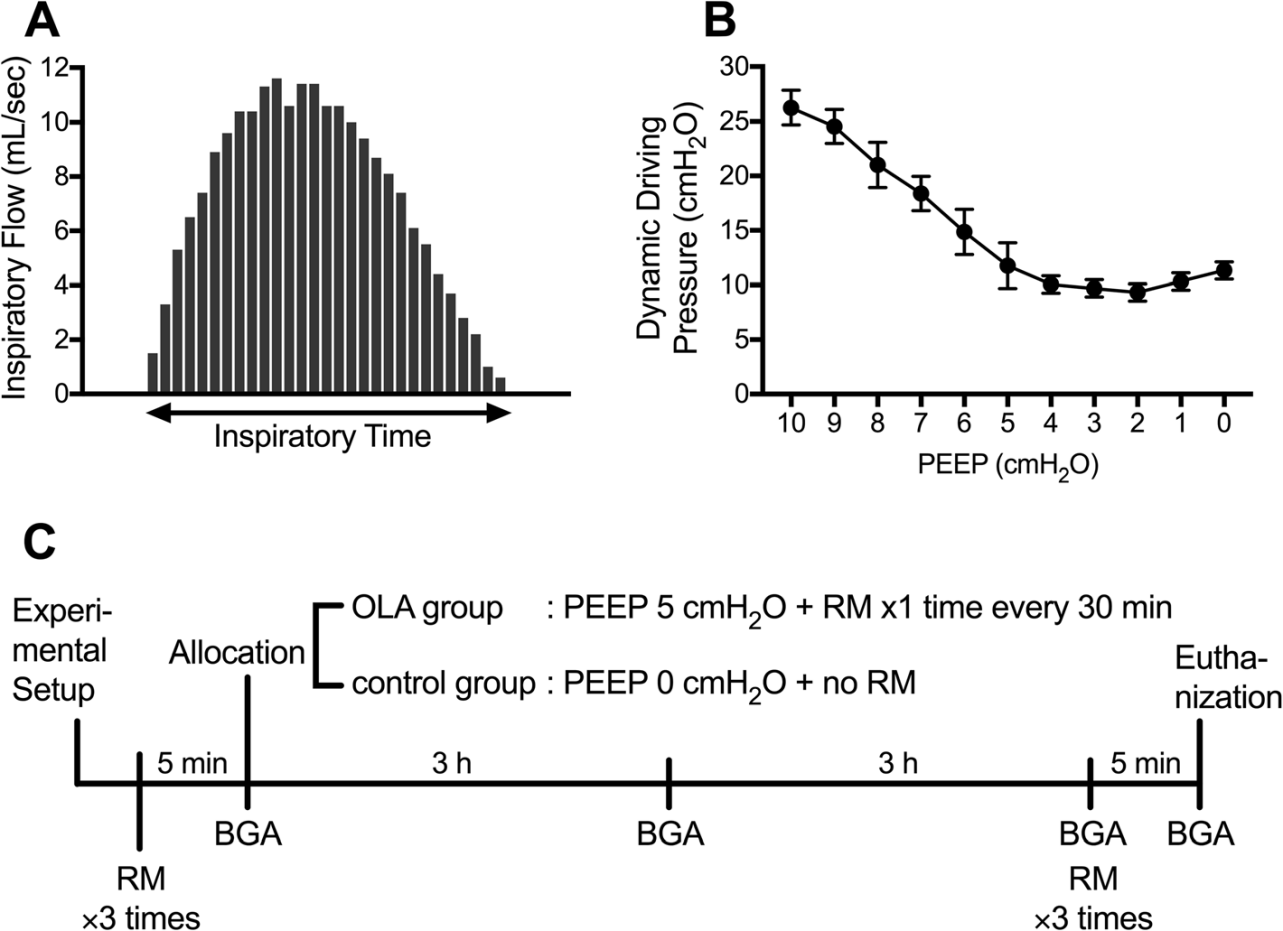
Inspiratory flow pattern, dynamic driving pressures during positive end-expiratory pressure (PEEP) titration, and the experimental scheme. **a** Inspiratory flow pattern of the small animal ventilator (SN-480-7, Shinano Seisakusho). **b** Changes in the dynamic driving pressures when decreasing PEEP from 10 to 0 cm H_2_O at intervals of 2 cm H_2_O. Data represent the means ± SD. **c** Schematic diagram of the experimental design. BGA, blood gas analysis; OLA, open lung approach; RM, recruitment maneuver

First, we recorded the inspiratory flow pattern of the small animal mechanical ventilator. Because the inspiratory flow from the ventilator is proportional to piston speed, we obtained videos of the piston movement with a digital video camera and analyzed the piston speed using ImageJ software. We confirmed that the inspiratory flow of the ventilator at end-inspiration was near zero (Fig. 1a). We therefore assumed that the peak inspiratory pressure was almost equal to the peak alveolar pressure (plateau pressure) and calculated the “dynamic” ΔP as the difference between the peak inspiratory pressure and PEEP.

Subsequently, we evaluated the association between dynamic ΔP and PEEP levels in rats administered with LPS (*n* = 3). After the RM, the dynamic ΔP was recorded while decreasing PEEP from 10 to 0 cmH_2_O at an interval of 2 cmH_2_O (Fig. 1b). Based on this observation, we set the PEEP levels in the animals managed with OLA at 5 cmH_2_O, which was derived by adding 2 cmH_2_O to the PEEP level that achieved the minimal dynamic ΔP.

In the main experiment (Fig. 1c), baseline physiological parameters were measured and arterial blood gas was analyzed 5 min after the RM. Thereafter, rats were randomly allocated to two experimental groups: the OLA and control groups. Twenty-two rats were used in the main experiment. One rat died before the experimental allocation. After the allocation, a rat in the control group died due to airway obstruction, and thus data from 20 animals were included in the final analysis (*n* = 10 per group). In the OLA group, 5 cmH_2_O PEEP was applied and the RM was performed every 30 min. Neither PEEP nor RM was applied to rats in the control group. The tidal volume was adjusted to maintain a dynamic ΔP of 15 cmH_2_O. In parallel, ventilation frequency was adjusted to maintain a constant minute volume.

After 6 h of mechanical ventilation, the RM was performed three times in both groups to inflate the reversible atelectatic lung region, because formation of atelectasis can often be misinterpreted as ventilator-induced-lung injury in rats [27]. After the final RM, the setting of the mechanical ventilation for both groups was changed to the initial setting as follows: tidal volume, 10 mL/kg; frequency, 65/min; and PEEP 5 cmH_2_O. At 5 min after the final RM, rats were euthanized by blood removal from the right carotid artery.

Arterial blood gas analysis was performed at the 3-h time point, and pre RM and 5 min post final RM. Broncho-alveolar lavage flood (BALF) was collected by lavaging the right lung with two separate 0.5-ml aliquots of PBS containing 0.6 mM EDTA. The left lung was fixed in paraformaldehyde and embedded in paraffin, as described previously [5], for histological analysis.

In another series of experiments, we measured esophageal pressures during mechanical ventilation. At the cervical incision for tracheostomy, the esophagus was identified and cannulated with a 20-gauge catheter filled with normal saline [28]. Then, the cannula was connected to a pressure transducer and esophageal pressures were recorded every hour. We performed an occlusion test to confirm that the cannula was located in the appropriate position [29]. In this experiment, 10 rats were randomly allocated to the OLA and control groups (*n* = 5 per group). Transpulmonary pressures (airway pressure – esophageal pressure) at end-expiration and end-inspiration and transpulmonary driving pressures (end-inspiratory transpulmonary pressure – end-expiratory transpulmonary pressure) were calculated.

### Analysis of BALF

Collected BALF was stained with Samson’s reagent solution (Nacalai Tesque, Kyoto, Japan). White blood cells in the BALF were counted using a cell counter plate. Thereafter, BALF was centrifuged at 1000 *g* for 10 min at 4 °C and aliquots of the supernatant were stored at − 80 °C for quantification of total protein and cytokines. Total protein concentration in the BALF was quantified by a bicinchoninic acid (BCA) method (Thermo Fisher Scientific, Waltham, MA, USA). Multiple cytokines and tissue injury markers in the BALF were analyzed using cytokine array assays (RayBiotech, Atlanta, GA, USA) following the manufacturer’s instruction. Then, proteins with significantly different levels between the two groups were quantified using the following ELISA kits according to the manufacturer’s instruction: ICAM-1: DY583 (R&D Systems, Minneapolis, MN, USA); RAGE: DY1616 (R&D Systems); CXCL-7: ERPPBP (Thermo Fisher Scientific).

### Histological examinations

The paraffin-embedded left lung sections were stained with hematoxylin and eosin as described previously [5]. Twelve fields were randomly selected from each of the dorsal or ventral lung areas (a total of 24 fields per animal), and were histopathologically evaluated in a blinded manner, following previously described methods [30].

### Computed tomography (CT)

A separate group of rats (*n* = 4 per group and time course) was used for CT evaluations. Either at the 3-h time point or 5 min after the final RM, rats were euthanized, and their tracheas were ligated at end-expiration. Immediately after euthanasia, a pulmonary CT image was obtained using a micro-CT imager (RIGAKU, Tokyo, Japan). The aerated lung volume was analyzed and calculated using ImageJ software.

### Statistical analysis

Data are presented as means ± standard deviations (SD). GraphPad Prism 6 (GraphPad Software, La Jolla, CA, USA) was used for all statistical analyses. Statistical significance was set at *p* < 0.05. Physiological parameters and lung histology scores were analyzed by two-way repeated-measures analysis of variance. Aerated lung volumes were analyzed by two-way analysis of variance. Post-hoc Sidak’s multiple comparison test was performed when there was a significant interaction effect. Cytokine array assay data were analyzed by multiple *t* test, using the false discovery rate approach with the two-stage step-up method of Benjamini, Krieger, and Yekutieli [31]. The false discovery rate was set at 5%. Protein concentrations, white blood cell counts, and ELISA data were analyzed by Welch’s *t* test.

## Results

### Physiological and mechanical ventilation parameters

**Fig. 2:**
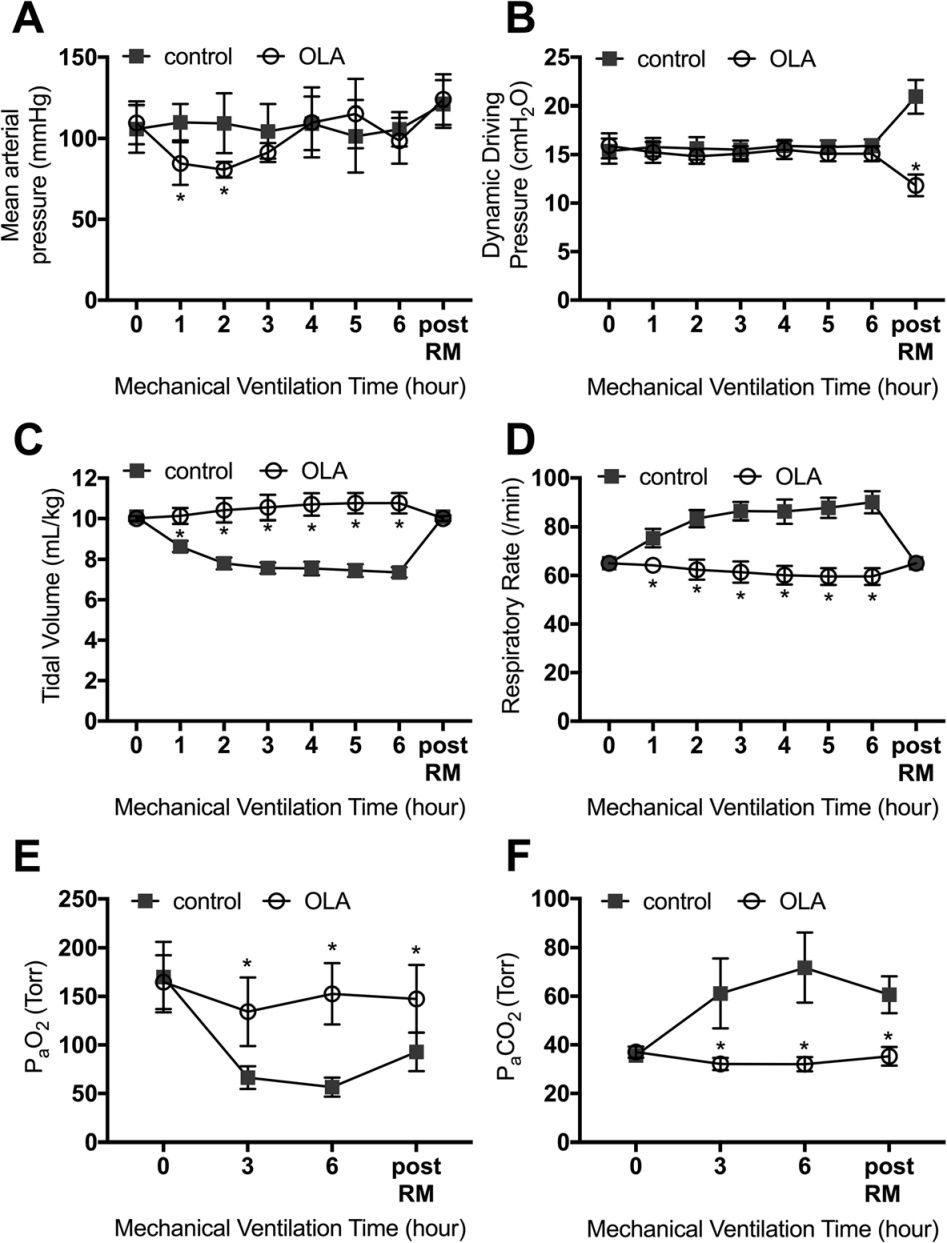
Physiological and mechanical ventilation parameters. **a** Mean arterial blood pressure. **b** Dynamic driving pressure. **c** Tidal volume. **d** Respiratory rate. **e** Partial pressure of arterial oxygen (P_a_O_2_). **f** Partial pressure of arterial carbon dioxide (P_a_CO_2_). *p < 0.05 vs. control group. Data represent the means ± SD. OLA, open lung approach; RM, recruitment maneuver

The mean arterial pressure (Fig. 2a) was lower in the OLA group at the 1-h and 2-h time-points of the experimental protocol than in the control group. However, mean arterial pressure was kept at over 70 mmHg in all the animals. There was no significant difference in mean arterial pressure between the two groups at time points after 3 h.

In both groups, the dynamic ΔP (Fig. 2b) was maintained at 15 cmH_2_O throughout the experimental protocol until the final RM. Both end-expiratory and end-inspiratory transpulmonary pressures were higher in the OLA group than in the control group, and transpulmonary driving pressures were kept at almost the same level between the two groups until the final RM (Additional file 1: Figure S1). Tidal volume (Fig. 2c) was significantly lower in the control group than in the OLA group, to maintain a constant dynamic ΔP. In parallel, the respiratory rate (Fig. 2d) was higher in the control group, to maintain the same minute ventilation volume. After the final RM, the tidal volume was set to the same value in both groups; dynamic ΔP was higher in the control group (20.9 ± 1.7 vs. 11.8 ± 1.1 cmH_2_O, *p* < 0.0001). Moreover, the end-inspiratory transpulmonary pressure (Additional file 1: Figure S1A) and transpulmonary ΔP (Additional file 1: Figure S1B) were significantly higher in the control group after the final RM (transpulmonary ΔP: 17.3 ± 3.3 vs. 9.7 ± 1.8 cmH_2_O, *p* < 0.0001). These results indicated that the lung compliance was lower in the control group, even after inflating the reversible atelectatic region.

The initial partial arterial pressure of oxygen (P_a_O_2_) (Fig. 2e) and partial arterial pressure of carbon dioxide (P_a_CO_2_) (Fig. 2f) values were not significantly different between the two groups. During the ventilation protocol, P_a_O_2_ was higher and P_a_CO_2_ was lower in the OLA group than in the control group, and the difference remained significant even after the final RM (P_a_O_2_, 92.8 ± 19.9 vs. 147.5 ± 34.9 mmHg, *p* = 0.0001, P_a_CO_2_, 60.6 ± 7.6 vs. 35.3 ± 3.8 mmHg, *p* < 0.0001).

### Lung CT

**Fig. 3:**
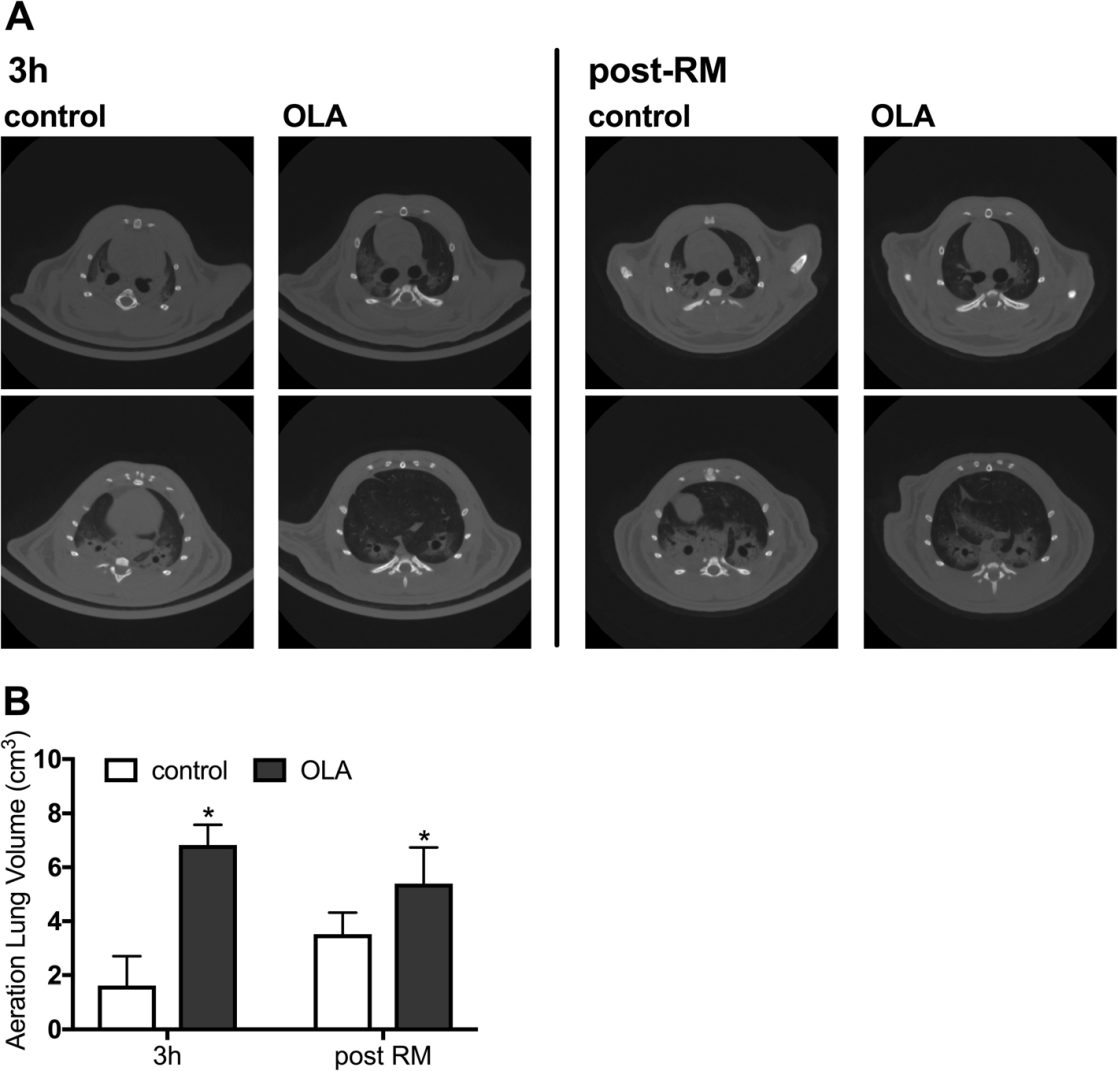
Computed tomography. **a** Representative pulmonary computed tomography images taken after 3 h of mechanical ventilation or after the final recruitment maneuver following 6 h of mechanical ventilation. **b** Aerated lung volumes calculated from computed tomography images. *p < 0.05 vs. control group. Data represent the means ± SD. OLA, open lung approach; RM, recruitment maneuver

At the 3-h time point of the experimental protocol and 5 min after the final RM, lung CT images were obtained at end-expiration. At the 3-h time point, atelectasis was observed particularly in the dorsal lung regions of the control group (Fig. 3a). At the same time, the measured volume of the aerated lung regions at end-expiration was significantly larger in the OLA group than in the control group (1.63 ± 1.08 vs. 6.83 ± 0.75 cm^3^, *p* < 0.0001) (Fig. 3c). After the final RM, atelectasis was reduced in the control group (Fig. 3b); however, the difference in the aerated lung volume between the OLA and control groups remained statistically significant (3.52 ± 0.80 vs. 5.40 ± 1.34 cm^3^, *p* = 0.0451) (Fig. 3c).

### Histological lung damage

**Fig. 4:**
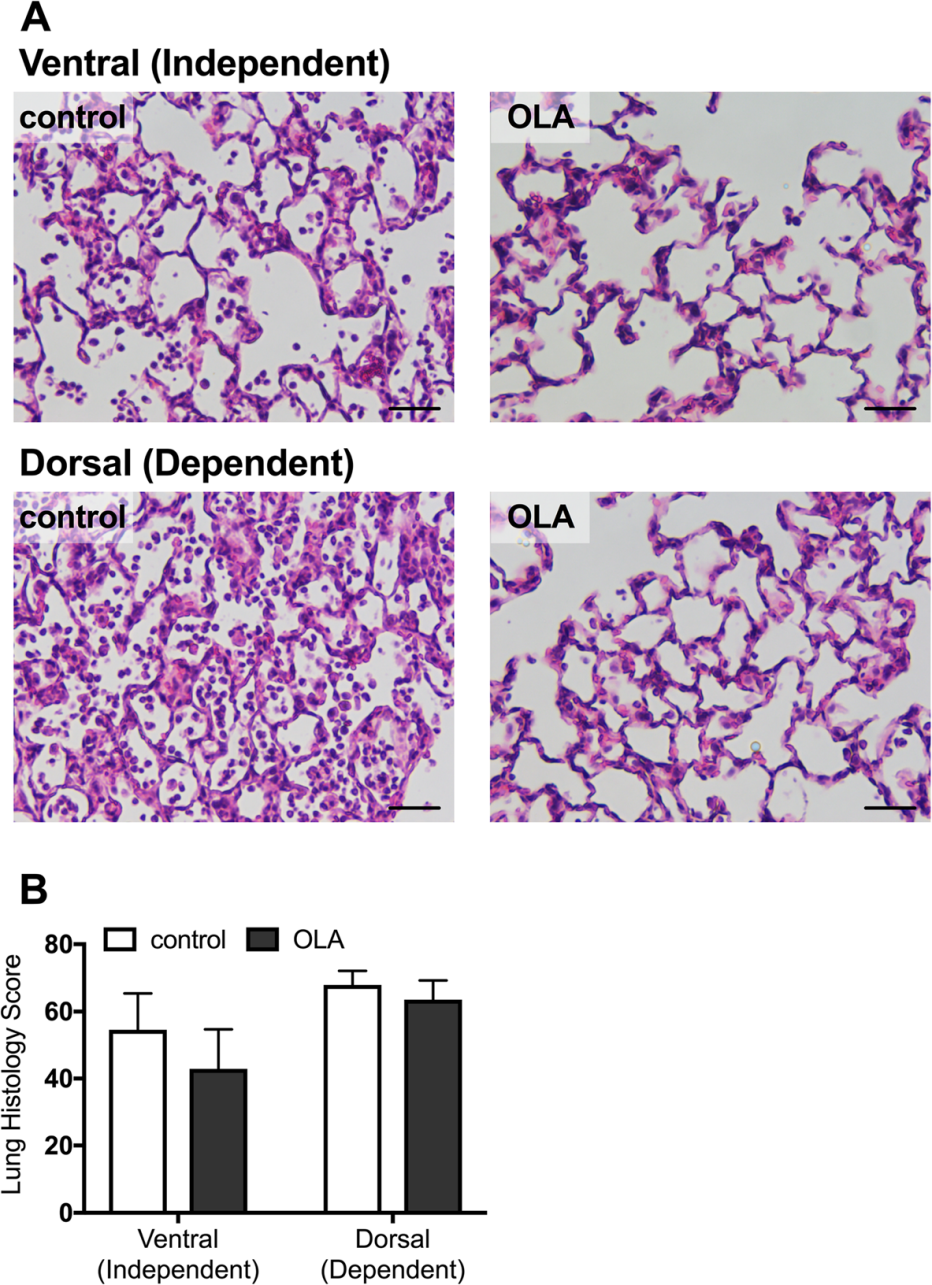
Histology. **a** Representative images of lung sections stained with hematoxylin and eosin. **b** Histological scores assessed in a blinded manner. Data represent the means ± SD. OLA, open lung approach

We next evaluated the histological lung damage in the ventral (independent) and dorsal (dependent) lung areas (Fig. 4). Lung histology scores were significantly higher in the dorsal dependent area than in the ventral area, and OLA attenuated the histological damage (effect of lung area, *F* = 85.63, *p* < 0.0001; effect of OLA management, *F* = 5.362, *p* = 0.0326; interaction effect, *F* = 3.908, *p* = 0.0636).

### Analysis of alveolar barrier injury and inflammatory mediators

**Fig. 5:**
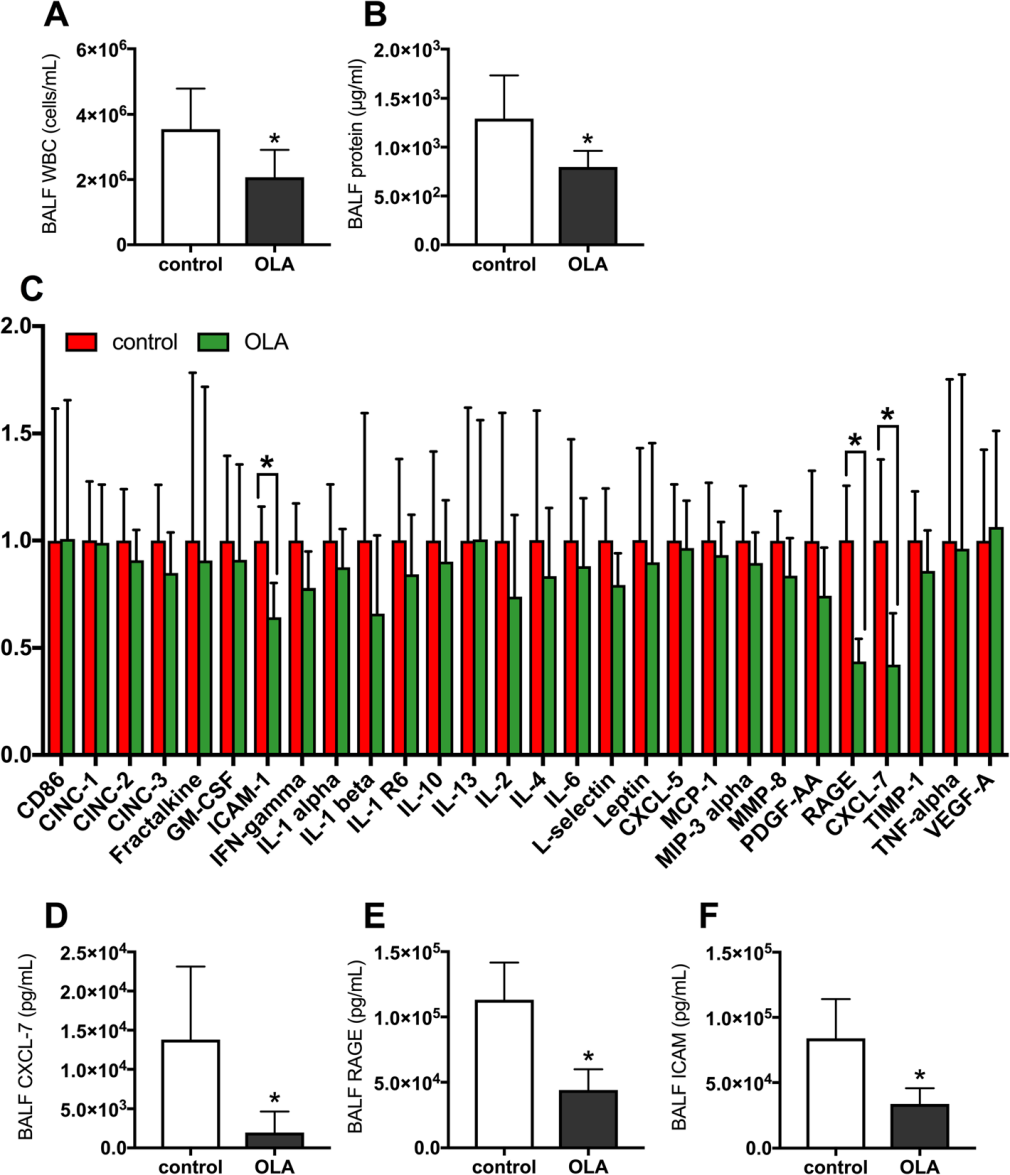
Analysis of bronchoalveolar lavage fluids (BALF). **a** White blood cell counts (WBC). **b** Protein concentrations. **c** Comprehensive analysis of inflammatory and tissue injury markers using cytokine array analysis. **d** Intercellular adhesion molecule-1 (ICAM-1), **e** soluble receptor for advanced glycation end products (RAGE), **f** CXC chemokine ligand-1 (CXCL-7) concentrations in the BALF quantified by ELISA. *p < 0.05 vs. control group. Data represent the means ± SD. OLA, open lung approach

In the OLA group, the number of white blood cells in the BALF was significantly lower than in the control group (354.9 ± 124.4 vs. 207.1 ± 84.3 × 10^4^ cells/mL, *p* = 0.0068) (Fig. 5a). Moreover, management with OLA significantly decreased the protein concentrations in BALF, which is an indicator of alveolar barrier disruption (1292.1 ± 438.0 vs. 796.7 ± 165.3 μg/mL, *p* = 0.0061) (Fig. 5b).

Next, we performed comprehensive analysis of inflammatory and tissue injury markers in BALF using cytokine array analysis (Fig. 5c). The concentration of CXCL7, a platelet-derived chemokine, was significantly lower in the OLA group than in the control group. Moreover, OLA decreased levels of RAGE and ICAM-1, indicators of alveolar epithelial and endothelial injury [32, 33], in BALF. We also confirmed that OLA decreased CXCL-7 (13,809 ± 9322 vs. 1969 ± 2682 pg/mL, *p* = 0.0029), RAGE (113,156 ± 28,580 vs. 44,048 ± 15,904 pg/mL, *p* < 0.0001), and ICAM-1 (83,995 ± 30,062 vs. 33,912 ± 11,796 pg/mL, *p* = 0.0004) levels in BALF, using ELISA (Fig. 5d−f). These results indicated that OLA protects the alveolar barrier by suppressing platelet activation and neutrophil infiltration into the alveoli.

## Discussion

In the present study, we demonstrated that OLA during mechanical ventilation has lung-protective effects against LPS-induced experimental ARDS, even when the dynamic ΔP was not decreased. In the previous experimental studies demonstrating the benefits of OLA, such as those using PEEP, a similar tidal volume was used irrespective of whether the animals received OLA [8, 20–26]. Therefore, it has remained unclear whether the protective effects are mediated by decreases in ΔP or by improvement in lung aeration per se. In the present study, to maintain a similar ΔP in both groups, the tidal volume was adjusted, and a significantly lower tidal volume was applied in the control group. Our results clearly demonstrated the driving pressure-independent benefits of OLA, which, to our knowledge, have not been reported previously.

Recently, Amato et al. have reported that ΔP is the ventilation variable that is most strongly associated with mortality in patients with ARDS [18]. Using mediation analysis, their report demonstrated that the application of PEEP seems to be beneficial only when the ΔP is lowered by the application of PEEP. Additionally, an experimental study utilizing a ventilator-induced lung injury model demonstrated that PEEP is protective only if associated with a reduced ΔP [19]. The results of these reports suggest that lung protection can be achieved by lowering ΔP, even without improvement of lung aeration. However, it is known that development of atelectasis per se enhances alveolar stress in the peri-atelectatic region [9, 10], and causes hypoxia-induced inflammation [5, 6] and pulmonary circulatory failure [7]. Our present results indicate that, in addition to lowering ΔP, maintaining lung aeration is important for lung protective management in ARDS.

In the present study, we applied both PEEP and RM as OLA. There are several methods to determine optimal PEEP during mechanical ventilation [34, 35]. We determined the PEEP level in the OLA group based on the lung mechanics required to maximize lung dynamic compliance [36]. Although there is no clear consensus as to the best approach for determining PEEP, the approach used in the present study is reported to exert lung protection with minimal adverse effects [37]. On the other hand, there are several conflicting experimental studies on RM [26, 38–41]; however, meta-analysis of clinical studies has indicated that RM in patients with ARDS may reduce intensive care unit mortality [14, 15]. Therefore, we believe there is some rationale for utilizing RMs as a part of OLA. Future studies should investigate the individual effects of RM and PEEP.

As expected, lung aeration, arterial oxygenation, and gas exchange were significantly better in the rats receiving OLA than in rats managed without OLA during the mechanical ventilation protocol. We finally performed the RM to ensure that we did not misinterpret the formation of atelectasis as lung damage [27]. In fact, in the control group, RM after 6 h of mechanical ventilation increased the aerated lung volume and improved arterial oxygenation, and decreased carbon dioxide levels; however, there were significant differences in these parameters between the OLA and control groups even after the RM. These results indicate that atelectasis not only causes oxygenation impairment, but also induces respiratory dysfunction that could not be easily reversed by RM.

Lung histologic analysis and analysis of BALF revealed that OLA attenuated neutrophil infiltration and alveolar barrier protein leakage. Moreover, levels of the alveolar epithelial and endothelial injury markers, RAGE and ICAM-1 [32, 33], in the BALF were significantly lower in the OLA group than in the control group. These findings also indicate that the atelectasis-induced respiratory dysfunction observed in the control group is not only an easily reversible functional problem, but is also accompanied by tissue injury. Although the observation period in the present study was only 6 h, our results show the suppression of alveolar tissue injury by OLA. Therefore, it is plausible that OLA would yield long-term clinical benefits, such as prolongation of ventilator-free days or improvement of mortality.

The comprehensive analysis of inflammatory cytokines revealed that OLA decreased the level of CXCL-7, a platelet-derived chemokine in the BALF. Hypoxia is a known cause of platelet activation [42]. Therefore, OLA might attenuate platelet activation by decreasing hypoxic atelectatic lung regions. Recently, the potential contribution of CXCL-7 to acute lung injury has been reported in a study using knock-out mice [43]. It is possible that suppression of CXCL-7 by OLA leads to decreased neutrophil infiltration into the alveolar space. Moreover, platelet activation might enhance pulmonary vascular thrombosis, which may in turn increase the dead space fraction, with CO_2_ retention, in the control group. As platelet activation and the increase in the platelet-derived CXCL-7 may be a potential therapeutic target for atelectasis-induced lung injury, further studies are warranted.

The arterial carbon dioxide level was significantly higher in the control group throughout the ventilation protocol. One of the possible reasons for CO_2_ retention was the increase in the relative dead space fraction due to the smaller tidal volume in the control group than in the OLA group. Another explanation is that alveolar hypoxia in the control group might have disturbed pulmonary blood flow and gas exchange through hypoxia-induced pulmonary vasoconstriction. Moreover, as mentioned above, hypoxia-induced platelet activation might also have disturbed the pulmonary circulation through thrombosis formation. Hypercapnia is known to exert anti-inflammatory effects [44–46] by suppressing NF-κB [47, 48]. Therefore, it is unlikely that the hypercapnia observed in the control group worsened the inflammation and tissue injury. On the other hand, hypercapnia has been reported as an unfavorable prognostic factor in pneumonia [49, 50] and in mechanically ventilated patients [51], possibly due to immunosuppression [52–55]. Thus, the improvement of gas exchange achieved by OLA may have advantages in terms of appropriate immunological responses against infection, which is the leading cause of ARDS.

The clinical efficacy of OLA has been evaluated in several studies. A recent meta-analysis has revealed that the high-PEEP strategy is not associated with mortality reduction, as compared with low-PEEP management [16]. Moreover, a recent randomized controlled trial, named Alveolar Recruitment for Acute Respiratory Distress Syndrome Trial (ART) [56], demonstrated that the management of ARDS patients with lung recruitment and titrated PEEP increased all-cause mortality, as compared to the low-PEEP strategy proposed by ARDSNet [57]. One of the potential explanations for this finding is that lung recruitability was low in the patients in the ART trial and that the harmful effects of OLA, such as circulatory failure, overdistension, or barotrauma, overcame its beneficial effects. In the present study, although the mean arterial pressure was significantly lower in the OLA group for the first 2 h of the ventilation protocol, lung aeration was significantly improved in the OLA group and no barotrauma was observed. The high lung-recruitability in the present study might have favorably influenced the effects of OLA. Additionally, the control group in the ART trial received substantial PEEP, whereas the control group in the present study was managed with zero PEEP. Although the present study suggests that lung aeration should be maintained to protect lungs against ARDS-related damage, it is also necessary to balance the beneficial and harmful effects of OLA. How to optimize OLA is a very important future focus for research in the mechanical ventilation of patients with ARDS.

The present study had some limitations. First, we applied only one level of ΔP to animals. We chose 15 cmH_2_O as the level of ΔP, because it seemed to be the upper limit of the safe ΔP level in patients with ARDS [17, 58], while a ΔP of less than 15 cmH_2_O could cause severe hypoxia and hypercapnia in the control group. However, it is unclear whether the protective effects of OLA would be observed when using a different level of ΔP. Second, we used only one animal model of ARDS, intratracheal LPS-induced ARDS, in the present study. In an ARDS model with a different etiology, such as systemic sepsis, the effects of OLA might be different. Third, management with zero PEEP in the control group is far from standard clinical practice. We used zero PEEP management as the control to recapitulate development of the marked atelectasis observed in patients with ARDS. However, this may limit the generalization of our results to clinical settings.

## Conclusions

In conclusion, OLA improved lung aeration and had protective effects against experimental ARDS, independent of ΔP. In addition to lowering ΔP, maintaining lung aeration is an important aspect of lung protective management in ARDS.

## Additional file

Additional file 1: **Figure S1.** Transpulmonary pressures. The data were obtained from a separate group of animals, different to those used in the main experiment. (A) End-inspiratory transpulmonary pressure. (B) End-expiratory transpulmonary pressure. (C) Transpulmonary ΔP. Transpulmonary pressures were calculated by subtracting esophageal pressures from airway pressures: *p < 0.05 vs. control group. Data represent the means ± SD. (TIFF 850 kb)

## Abbreviations

ARDS: Acute respiratory distress syndrome
BALF: Broncho-alveolar lavage fluid
CT: Computed tomography
CXCL-7: CXC chemokine ligand-1
ELISA: Enzyme-linked immunosorbent assay
ICAM-1: Intercellular adhesion molecule-1
OLA: Open lung approach
P_a_CO_2_: Partial arterial pressure of carbon dioxide
P_a_O_2_: Partial arterial pressure of oxygen
PBS: Phosphate-buffered saline
PEEP: Positive end-expiratory pressure
RAGE: Receptor for advanced glycation end products
RM: Recruitment maneuver
