# Supplementary figures and images for: Driving-pressure-independent protective effects of open lung approach against experimental acute respiratory distress syndrome
Source: Crit Care. 2018 Sep 23;22:228. doi: 10.1186/s13054-018-2154-2 (PMC6151188; doi:10.1186/s13054-018-2154-2)

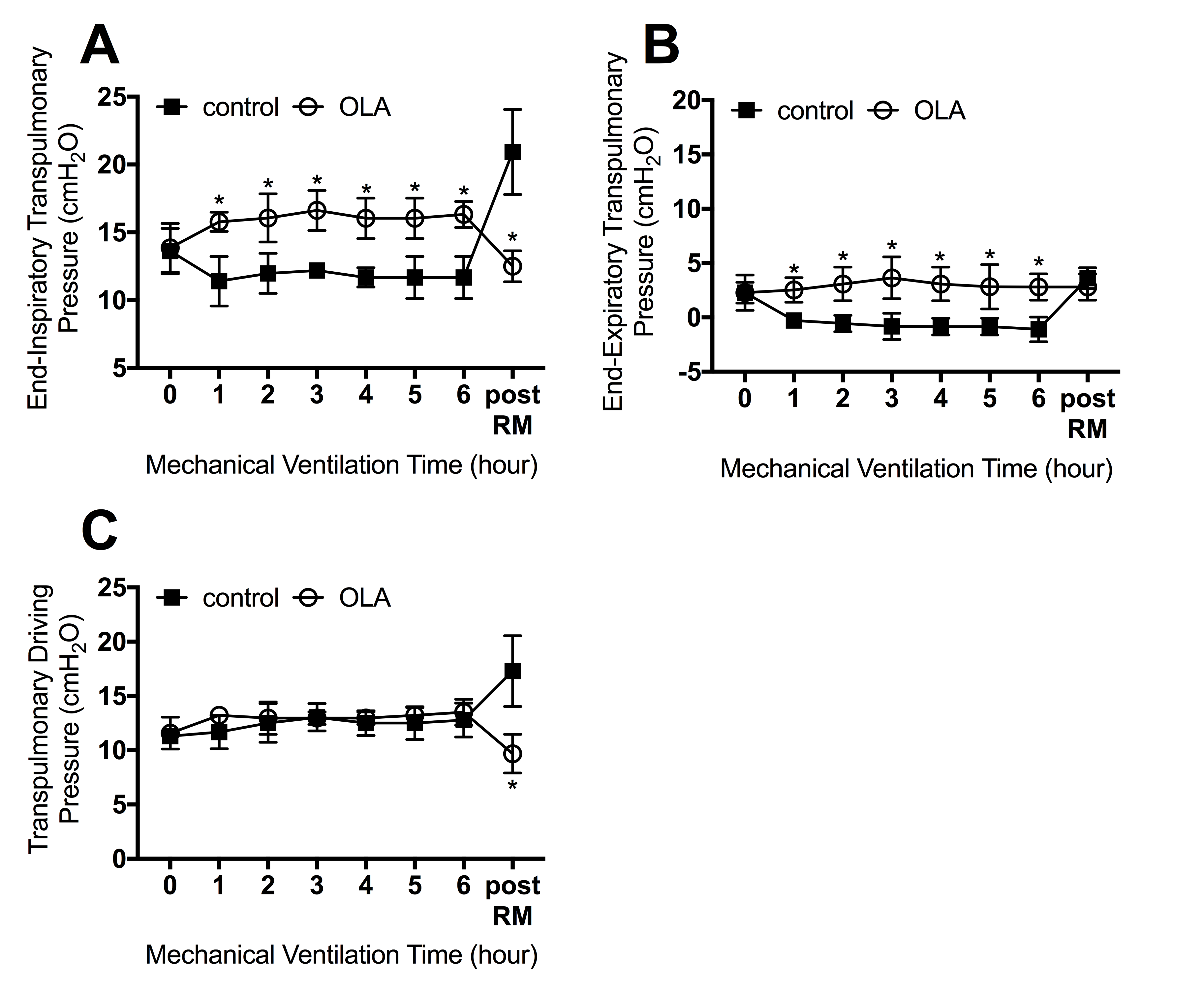

Supplement: Supplementary file 1 — Figure S1. Transpulmonary pressures. The data were obtained from a separate group of animals, different to those used in the main experiment. (A) End-inspiratory transpulmonary pressure. (B) End-expiratory transpulmonary pressure. (C) Transpulmonary ΔP. Transpulmonary pressures were calculated by subtracting esophageal pressures from airway pressures: *p < 0.05 vs. control group. Data represent the means ± SD. (TIFF 850 kb) [file 13054_2018_2154_MOESM1_ESM.tiff]
